# Using Nuclear Receptor Activity to Stratify Hepatocarcinogens

**DOI:** 10.1371/journal.pone.0014584

**Published:** 2011-02-14

**Authors:** Imran Shah, Keith Houck, Richard S. Judson, Robert J. Kavlock, Matthew T. Martin, David M. Reif, John Wambaugh, David J. Dix

**Affiliations:** National Center for Computational Toxicology, Office of Research and Development, United States Environmental Protection Agency, Research Triangle Park, North Carolina, United States of America; Universität Heidelberg, Germany

## Abstract

**Background:**

Nuclear receptors (NR) are a superfamily of ligand-activated transcription factors that control a range of cellular processes. Persistent stimulation of some NR is a non-genotoxic mechanism of rodent liver cancer with unclear relevance to humans. Here we report on a systematic analysis of new *in vitro* human NR activity data on 309 environmental chemicals in relationship to their liver cancer-related chronic outcomes in rodents.

**Results:**

The effects of 309 environmental chemicals on human constitutive androstane receptors (CAR/NR1I3), pregnane X receptor (PXR/NR1I2), aryl hydrocarbon receptor (AhR), peroxisome proliferator-activated receptors (PPAR/NR1C), liver X receptors (LXR/NR1H), retinoic X receptors (RXR/NR2B) and steroid receptors (SR/NR3) were determined using *in vitro* data. Hepatic histopathology, observed in rodents after two years of chronic treatment for 171 of the 309 chemicals, was summarized by a cancer lesion progression grade. Chemicals that caused proliferative liver lesions in both rat and mouse were generally more active for the human receptors, relative to the compounds that only affected one rodent species, and these changes were significant for PPAR (p
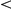
0.001), PXR (p
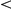
0.01) and CAR (p
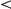
0.05). Though most chemicals exhibited receptor promiscuity, multivariate analysis clustered them into relatively few NR activity combinations. The human NR activity pattern of chemicals weakly associated with the severity of rodent liver cancer lesion progression (p
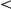
0.05).

**Conclusions:**

The rodent carcinogens had higher *in vitro* potency for human NR relative to non-carcinogens. Structurally diverse chemicals with similar NR promiscuity patterns weakly associated with the severity of rodent liver cancer progression. While these results do not prove the role of NR activation in human liver cancer, they do have implications for nuclear receptor chemical biology and provide insights into putative toxicity pathways. More importantly, these findings suggest the utility of *in vitro* assays for stratifying environmental contaminants based on a combination of human bioactivity and rodent toxicity.

## Introduction

Nuclear receptors (NR) are a superfamily of ligand-activated transcription factors that regulate a broad range of biological processes including development, growth and homeostasis. NR ligands include hormones [1] and lipids [2] but also xenobiotics [3]. We are interested in NR because of their involvement in non-genotoxic rodent liver cancer [4], a frequently observed effect in chronic toxicity testing [5] and often a critical effect in risk assessments of chemicals. Inferring the risk of chemical-induced human liver cancer from rodent studies is difficult because the underlying mechanisms are poorly understood. Persistent activation of NR is believed to be a possible mode of action [6], [7] operative in various pathways leading to cancer [8]. This raises a public health concern because some environmental chemicals are human NR activators and non-genotoxic rodent hepatocarcinogens including: pesticides [9], [10], persistent chemicals [11], and plastics ingredients [6]. In addition, there is very little available biological information for thousands of environmental chemicals so that new tools are needed to characterize their potential for toxicity [12]–[15].

We are generating human *in vitro* NR assay data for hundreds of environmental chemicals as a part of the ToxCast project [15]. Most of the Phase I ToxCast chemicals have undergone long-term testing experiments in rodents and their chronic hepatic effects have been curated and made publicly available in the Toxicology Reference Database (ToxRefDB) [5]. Although small sets of chemicals have been evaluated using selected NR in the past, ToxCast is the largest public data set on chemicals, encompassing concentration-dependent NR activity and chronic outcomes including liver cancer. Hence, these data provide a unique opportunity to investigate relationships between *in vitro* NR activation and rodent hepatic effects.

Our objective is to stratify chemicals based on their putative mode of action for human toxicity using data ranging from *in vitro* molecular assays to *in vivo* rodent outcomes from ToxCast [16] and other available resources. We have previously evaluated supervised machine learning approaches [17] and used them to classify chemicals by chronic toxicity outcomes using *in vitro* data. In this analysis we used an unsupervised multivariate analysis of NR activities and rodent liver lesions to investigate a potential mode of action for non-genotoxic hepatocarcinogenesis.

## Results

### Nuclear Receptor Activity

Human NR activity for 309 environmental chemicals was obtained from in vitro high-throughput screening (HTS) experiments. Duplicates and triplicates for eight chemicals were included for quality control purposes. HTS data were collected for 10 out of the 48 human NR, selected based on availability of assays and potential relevance to toxicology, including: members of the NR1, NR2, NR3 and NR4 subfamilies. The aryl hydrocarbon receptor (AhR) data was also included because of its potential role in xenobiotic metabolism and non-genotoxic liver cancer [18]. A total of 54 HTS assays were used to interrogate different facets of receptor activation including: ligand binding in a cell-free system (Cell-free HTS); reporter gene activation in HEK293 human cells [19] (Cell-based HTS); multiplexed cis-activation and trans-activation assays for transcription factors in human HepG2 cells [20] (Multiplexed Transcription Reporter); and, multiplexed gene expression assays of xenobiotic metabolizing enzymes regulated by specific NR in primary human hepatocytes (Multiplexed Gene Expression). Data for chemical-assay pairs were collected in concentration-response format and either the AC50 concentration or the Lowest Effective Concentration (LEC) were reported (additional details are provided in supplementary methods, Text S1).

### Aggregate Nuclear Receptor Activity

**Figure 1:**
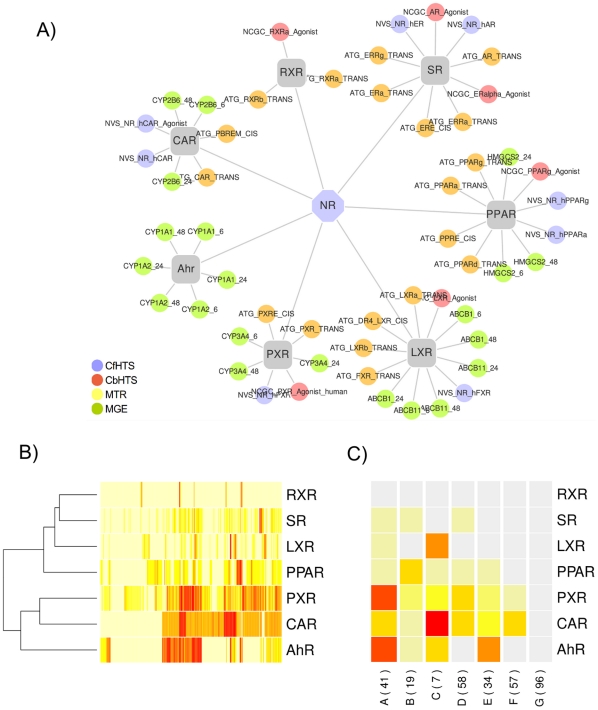
Nuclear receptor activity. Panel (a). Aggregation of 54 ToxCast assays for calculating seven nuclear receptor activities for AhR, CAR, PXR, PPAR, LXR, SR and RXR. Abbreviations for different types of assays described in the text. Panel (b). Nuclear receptor activities (rows) of 309 chemicals (columns). The color of each cell signifies degree of activity: gray means no activity, yellow is the least active and red the most active. The similarity between 7 nuclear receptor activities shown as a dendrogram on the left. Panel (c). Chemical nuclear receptor activity groups shown in columns labeled A-G and corresponding group size in parentheses. Colors represent relative activity of chemicals in each nuclear receptor activity group across rows: gray is minimal, yellow is the least and red the most.

To summarize the activity of chemicals across the NR superfamily we aggregated the ToxCast assays for genes and NR groups as follows: retinoic X receptor-like (RXR; 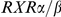; NR2B); peroxisome proliferator-activated receptor-like (PPAR; 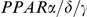; NR1C); constitutive androstane receptor (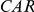; 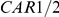; 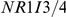); pregnane X receptor (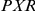; 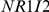); liver X receptor-like (LXR; 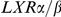, FXR; NR1H); and steroid receptor-like (SR; 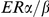, 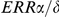, 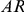). These are shown visually in Figure 1(a). As there were differences in the number and types of assays for each group, aggregate activity was calculated as the average potency across the assays measured by the AC50 or LEC (described in Methods). This approach aggregated NR binding, activation, agonism or antagonism results into a single assessment of activity.

The aggregate activity of each of 309 chemicals was calculated across all assayed NR with the results visualized as the heatmap in Figure 1(b). In this visualization, the rows represent the NR: RXR, LXR, AhR, SR, PPAR, CAR and PXR. Columns correspond to chemicals. The value of each cell is the aggregate scaled activity of a chemical-NR pair, and the column intensities signify the aggregate NR activity profile for each chemical (see Methods). The intensity of the colors signifies the degree of activity, where gray is inactive, yellow is the least active and red the most active. The dendrogram to the left of the NR shows their functional similarity across all 309 chemicals as two main groups. The first group contains CAR and PXR, which are most similar in their response across the chemicals, followed by AhR. The second group includes PPAR, LXR, SR and RXR. The descending order of similarity between: CAR, PXR, PPAR and SR is consistent with receptor homology. CAR and PXR are members of NR1I (thyroid hormone receptor-like), PPAR includes members of NR1C (peroxisome proliferator-activator receptor), SR represents subfamily NR3 (steroid receptor-like; estrogen and androgen). On the other hand, the activities of RXR are not similar to other NR1 members and AhR belongs to the basic Helix-Loop-Helix/Per/Arnt/Sim (bHLH-PAS) superfamily, which is distinct from NR.

### Combinatorial Nuclear Receptor Activity

The chemicals were clustered by similarity of aggregate NR activity into 7 putative groups (A-G) (described in Methods). The average activity profile of the NR groups (NRG) are shown in the columns of Figure 1(c). The rows signify the NR and their order from top to bottom shows decreasing promiscuity and potency. The letters and numbers in parentheses below each column are the cluster designation and the number of chemicals in each cluster, respectively. The colors signify the activity of a NR across the NRG: red shows consistent activity and yellow inconsistent activity. For example, the first column from the left of the heatmap shows NRG A, which contains 41 chemicals that tend to activate AhR, PXR, CAR, PPAR and in some cases also SR or LXR. These results concisely describe how the 309 chemicals and 54 molecular assays can be summarized by different groups of combinatorial NR activity. The NRG correctly grouped 6 out of the 8 replicate chemicals (Table 1). For the remaining two chemicals, the duplicate Dibutyl phthalate samples had low NR activity and grouped closely in NRG F and NRG G (these samples were separately sourced substances from two different vendors). The triplicate Prosulfuron samples did not group correctly and further analysis revealed this to likely be due to degradation of the parent chemical prior to conducting the assays.

**Table 1:** Chemicals grouped by nuclear receptor activity and lesion progression.

	A	B	C	D	E	F	G
I	Fludioxonil	Diclofop-methyl		Diethylhexyl	Carbaryl	Isoxaflutole	2,5-Pyridinedicarboxylic- acid, dipropyl ester
	Lactofen	Diclofop-methyl		phthalate			Pymetrozine
	Oxadiazon	Diclofop-methyl					Tepraloxydim
		Imazalil					
		Malathion					
		Vinclozolin					
II	Bensulide	Fentin		Buprofezin	Fenamidone	Butafenacil	Clodinafop-propargyl
	Bensulide	Fluazinam		Fenarimol		Diphenylamine	Pyrithiobac-sodium
	Bensulide	Spirodiclofen		Fluthiacet-methyl		Fenoxycarb	
	Dithiopyr			Piperonyl butoxide			
	MGK			Pyraflufen-ethyl			
	Triflumizole			Resmethrin			
III	Indoxacarb	Bromoxynil		Lindane	Clofentezine	Dicofol	3-Iodo-2-propynylbutylcarbamate
	Iprodione			Permethrin	Cyproconazole	Difenoconazole	3-Iodo-2-propynylbutylcarbamate
	Linuron			Prochloraz		Nitrapyrin	Dazomet
	Propiconazole			Propyzamide			Fenoxaprop-ethyl
	Thiazopyr						Fenoxaprop-ethyl
							Folpet
							Quizalofop-ethyl
							Thiamethoxam
IV	Isoxaben	Cinmethylin		Hexythiazox	Benfluralin	Maneb	2-Phenylphenol
	Methidathion				Benomyl	Primisulfuron-	Acephate
	Triadimefon				Bifenazate	Propoxur	Amitraz
	Triadimenol				Bromacil	Terbacil	Bentazone
	Tribufos				Fenitrothion		Cloprop
					Norflurazon		Daminozide
					Thiophanate-methyl		Dimethoate
					Triflusulfuron-methyl		Thiodicarb
V		Cyclanilide	Tebufenpyrad	Ametryn	Acetochlor	Dichlobenil	Mevinphos
				Dimethenamid	Simazine		
VI	Tetraconazole			Azoxystrobin	Boscalid	Oxasulfuron	Clothianidin
				Butachlor	Propanil	Sethoxydim	thephon
				Chlorpropham	Pyrimethanil	Tralkoxydim	Famoxadone
				Flufenacet			Rimsulfuron
				Pendimethalin			Thiram
				Quintozene			Trichlorfon
VII	Flutolanil			Bisphenol A	Carboxin	2,4-DB	Acetamiprid
	Oxyfluorfen			Carfentrazone-ethyl	Dichloran	Butylate	Asulam
	Triticonazole			d-cis,trans-Allethrin	Diuron	Chlorpyrifos-methyl	Azamethiphos
				Fipronil		Clorophene	Cymoxanil
				Metalaxyl		Fenbuconazole	Hexazinone
				Prallethrin		Flufenpyr-ethyl	Mesosulfuron-methyl
				Prosulfuron		Flumiclorac-pentyl	Novaluron
				Sulfentrazone		Fluoxastrobin	Prosulfuron
				Trifloxystrobin		Myclobutanil	Thiacloprid
						Prometon	Thidiazuron
						Prosulfuron	
						Tefluthrin	
						Triasulfuron	
VIII	Cyprodinil		Dimethomorph	Alachlor	Acibenzolar-S-Methyl	Emamectin benzoate	2,4-Dichlorophenoxyacetic acid (2,4-D)
	Etoxazole			S-Bioallethrin	Picloram	Icaridin	Chlorsulfuron
	Flumetralin			Ethofumesate	Thiabendazole	Paclobutrazol	Chlorsulfuron
	Hexaconazole			Flusilazole		Penoxsulam	Cyhalofop-butyl
	Methoxyfenozide			Fosthiazate		Triclosan	Dichlorprop
	Phosalone						Dichlorvos
	Pyraclostrobin						Mesotrione
	Tebufenozide						

Chemicals assigned to nuclear receptor groups (columns) and lesion progression groups (rows).

### Comparing NR Activity with Cancer Lesion Progression

*In vivo* rat and mouse long-term histopathology outcomes for chemicals were gathered from ToxRefDB [5] and organized by severity of lesions progressing to cancer. Of the 309 ToxCast chemicals, 232 were tested in 2-year chronic feeding studies in both rat and mouse, and were characterized by liver histopathology as follows: 61 caused no observable effects and 171 chemicals caused a range of lesions of varying severity.

The 61 chemicals negative for any liver injury include: Ethalfluralin, Fenamiphos, Fenthion, Isazofos, and Propetamphos (NRG A); Cyazofamid and Fenhexamid (NRG B); Fenpyroximate, Rotenone, Tebupirimfos (NRG C); and (51/61) in NRG D, E, F and G (see Dataset S4). Since the absence of rat or mouse liver toxicity is unusual after sustained treatment with a chemicals for two years, it can indicate an insufficient treatment dose (among other factors). When we reviewed the treatment protocols for these 61 chemicals we found that 7/10 chemicals in NRG A, B and C may have been administered at insufficient doses to produce hepatic effects. For example, Rotenone is a potent mitochondrial inhibitor and commonly used as a pesticide. It can cause rodent gastrointestinal injury at roughly 150 parts per million (ppm), however, it was only tested at a maximum dose of 3.75 ppm in the chronic study. Hence, we could not be certain about the absence of liver toxicity for these 61 chemicals despite a lack of nuclear receptor activity in a majority of 51 cases.

### Lesion Progression and Nuclear Receptor Activities

**Figure 2:**
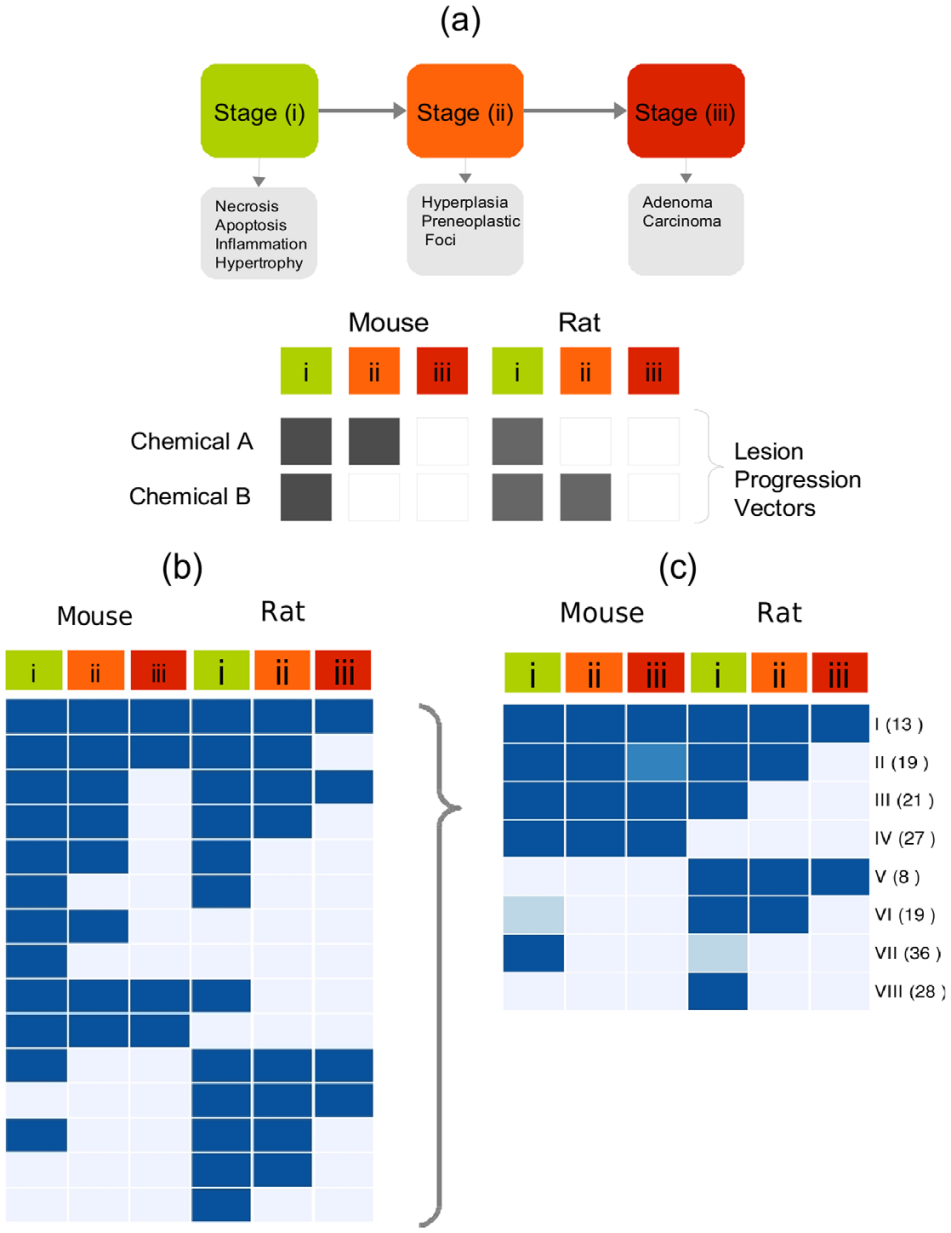
Cancer lesion progression. Panel (a). Chronic liver toxicity represented on the basis of cancer lesion progression as three histopathologic stages. Chronic toxicity testing results for each chemical across mouse and rat species are represented by six dimension lesion progression vector. Panel (b). Unique lesion progression vectors for all 171 chemicals. Columns represent histopathologic stages, and rows are groups of chemicals with unique combinations of lesions across the two species. Cell colors indicate presence (dark blue) or absence (light blue) of lesions. Panel (c). Chemical lesion progression groups in rows I-VIII and corresponding group sizes in parentheses. The proportion of chemicals in lesion progression groups producing lesions at a specific stage (column) are shown as color intensity of cells.

**Figure 3:**
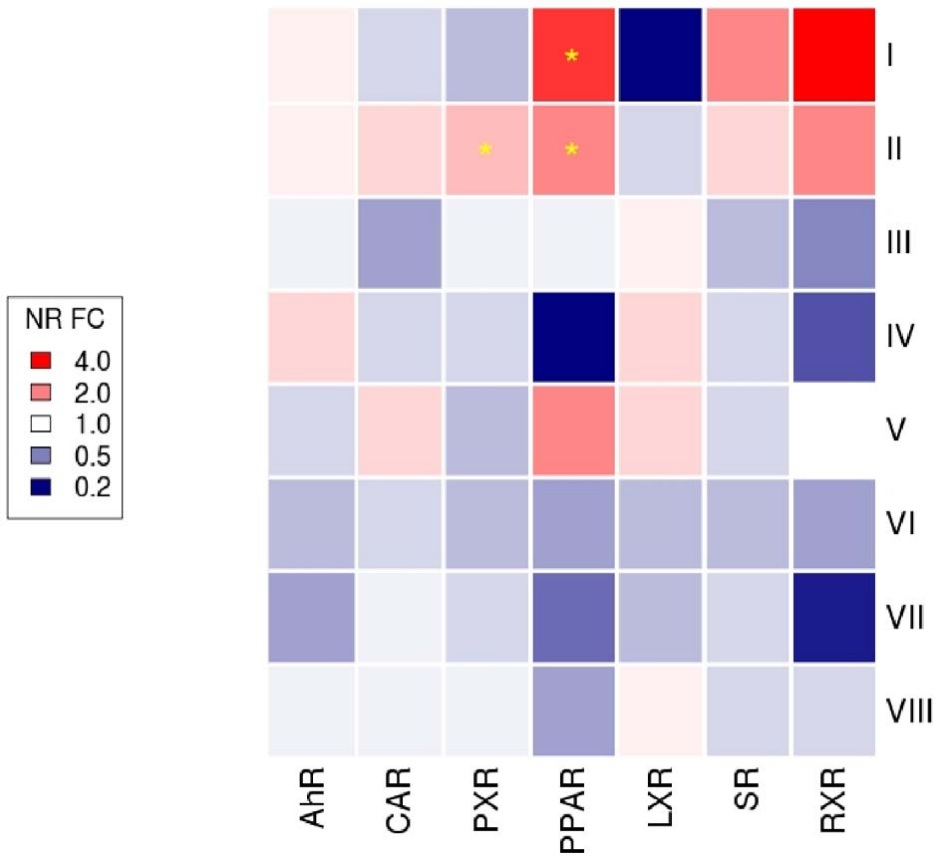
Nuclear receptor activity and cancer lesion progression. Visualizing the relationship between the aggregate nuclear receptor activities across the lesion progression group as a heatmap. The rows of the heatmap signify the lesion progression groups I-VIII and the columns show the aggregate nuclear receptor activities. The colors represent the ratio of the aggregate nuclear receptor activity between chemicals in a lesion progression group compared to others: decreased activities are shown in blue, no changes are shown in white and increased activity is shown in red. Statistically significant changes are shown with a yellow asterisk in the cell.

We assumed that dose selection was not an issue for the 171 chemicals that produced at least some liver toxicity in chronic rodent testing. Out of these 171 chemicals, 66 were mild hepatotoxicants, 43 produced different grades of proliferative lesions in rat and mouse, and 13 chemicals caused neoplastic lesions in both species. The severity and concordance of hepatic lesions across these 171 chemicals were clustered by similarity into eight lesion progression groups shown in Figure 2(c) (see Methods). The aggregate NR activities were systematically compared across all lesion progression groups (LPG) and visualized in Figure 3. The rows in Figure 3 correspond to the eight lesion progression groups (LPG I, II, III, IV, V, VI, VII, VIII) shown in Figure 2(c), and the columns are the NR: AhR, CAR, PXR, PPAR, LXR, SR, RXR. Each cell in the heatmap shows the ratio of the mean NR activities of chemicals in a LPG compared to all other LPG. The statistical significance of differences in mean NR activity was evaluated by permutation and corrected for multiple testing (see Methods). AhR, PPAR, SR and RXR showed 9% to 250% higher average activity for chemicals in LPG I as compared to the other chemicals but only PPAR showed a statistically significant (p
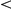
0.001) increase of 150%. For LPG II chemicals, all NR showed some increased activity except LXR, but only PPAR and PXR had statistically significant (p
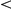
0.05) increases in activity of 80% and 50%, respectively. There were no statistically significant differences in NR activities for chemicals that produced only mouse proliferative lesions, however, the subset of mouse carcinogens showed a 30% increase in AhR activity but a 30% decrease in PPAR activity. Chemicals that produced only rat hepatic neoplasms had a 75% increase in PPAR activity, 23% increase in CAR activity and 30% increase LXR activity but none were statistically significant.

### Lesion Progression and Nuclear Receptor Activity Groups

**Figure 4:**
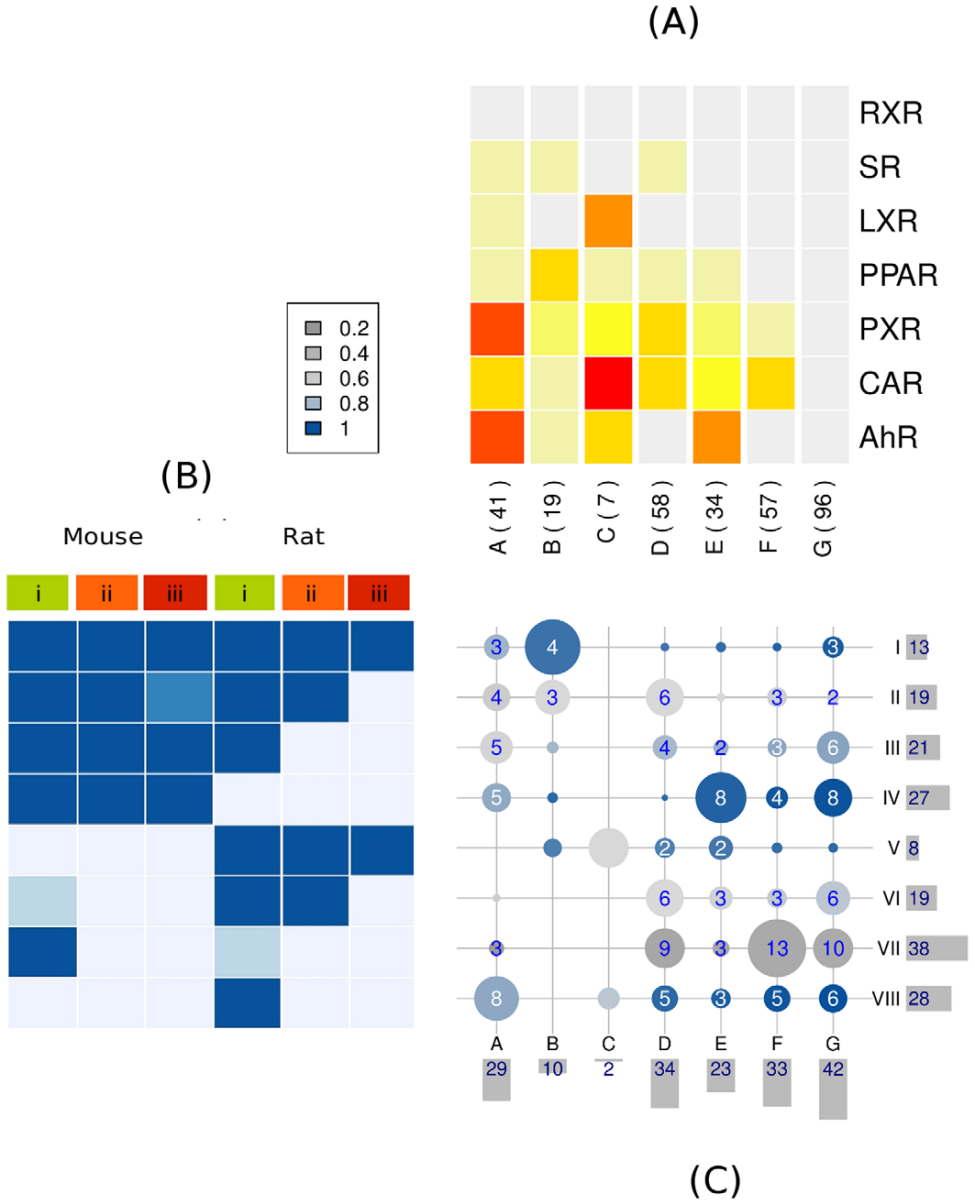
Relating nuclear receptor activity and cancer lesion progression. Panels 3(a) and (b) are taken from Panels 1(c) and 2(c), respectively. Panel 3(c). Visualizing the relationship between lesion progression group I-VIII (rows) and nuclear receptor groups A-G (columns). The proportion of chemicals in the intersection of lesion progression groups and nuclear receptor groups visualized by circle size. Confidence in chemical assignments to groups represented by color intensity from blue (high) to gray (low). Labels on the far right (I-VIII) and bottom (A-G) identify lesion progression group and nuclear receptor group, respectively. Bar plots on the far right and bottom indicate number of chemicals in each lesion progression group and nuclear receptor group, respectively.

The comparison between the LPG and NRG between 171 chemicals is visualized in Figure 4(c). The rows in Figure 4(c) are the eight lesion progression groups (LPG I, II, III, IV, V, VI, VII, VIII) shown in Figure 4(b) and the columns are the seven NR activity groups (NRG A, B, C, D, E, F, and G) shown in Figure 4(c). Each circle represents chemicals that have similar human NR activity and degree of rodent lesion progression. The size of each circle visualizes the proportion of chemicals across the LPG (rows) and NRG (columns), while the color signifies confidence in assignment of chemicals to each group (see Methods).

We designate each joint group by concatenating the identifier as: LPG-NRG, and interpret the first row of Figure 4(c), which corresponds to LPG I. The first circle from the left represents group I-A, which is formed by the intersection of 13 chemicals in LPG I and 29 chemicals in NRG A. The three chemicals in I-A (shown in the first row and first column of Table 1), fludioxonil, lactofen and oxadiazon were consistently active with AhR, PXR and CAR, but less frequently with PPAR, LXR and SR. Oxadiazon is a herbicide with known human PXR activity [21]. Lactofen is a poly-phenyl herbicide with 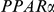 activity in mice [22]. Similarly, I-B, the second circle from the left, is the intersection of 13 chemicals in LPG I and 10 chemicals in NRG B. Chemicals in I-B include diclofop-methyl (each of the three replicates), imazalil, malathion and vinclozolin. These chemicals were most consistently active with PPAR, but also showed some activity with PXR, CAR, AhR and SR. Imazalil is an imidazole fungicide that perturbs human genes regulated by 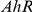 [23] and is also a 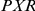 activator [21]; malathion is an organophosphorus pesticide with known SR activity [24]; vinclozolin, a dicarboximide fungicide is also a known SR active [25]; and diclofop-methyl has been shown to be PPAR [26] active in rats. Group I-D only contains diethylhexylphthalate (DEHP), which is a key plastics monomer, and has been shown to activate 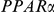 [27], 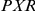 [28], and 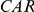 [29]. In group I-E we have carbaryl, which is a carbamate insecticide with 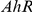 [30] and SR [31] activity. Lastly, chemicals in I-F and I-G had negligible NR activity, which could suggest that they act through other pathways.

Chemicals in LPG II produced only putative pre-neoplastic liver lesions in rat and mouse but there is limited prior knowledge about their NR activities.

LPG III only contains mouse hepatocarcinogens predominantly active with AhR, PXR and CAR, but some propensity for PPAR, LXR and SR. In III-A, the dicarboximide fungicide, iprodione, has been shown to activate 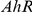 in human HepG2 cells [32]; linuron activates 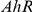 in mouse [33], 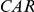 in rat [34], and the triazole fungicide, propiconazole, activates 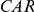, 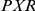 and 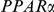 in mice [35]. The four chemicals in III-D namely, permethrin, lindane, prochloraz and propyzamide, are most consistently active for CAR, followed by AhR and PXR. In hepatocytes, permethrin [36] and lindane [37] induce expression of the 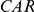, 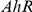 and 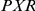 target xenobiotic metabolizing enzymes (XME), 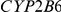, 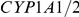, and 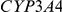, respectively. Prochloraz has only been observed to activate 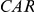 and 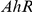 [37]. Chemicals in III-E have lesser overall NR activity but are generally more active with AhR and to a lesser degree with CAR, PXR and PPAR. One of the chemicals in III-D, cyproconazole, has been shown to induce expression of a cytochrome P-450 in the 2B subfamily (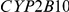), an XME regulated by 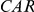 across different mouse strains [38], however, the expression of 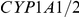 was not measured in this study.

The relationship between NR activation and cancer lesion progression is visualized by the location and size of circles: when the NR activity is greatest (NRG A), many of the chemicals are rodent hepatocarcinogens (LPG I) or just mouse carcinogens (LPG I-IV); and when NR activity is the least (NRG G), most of the chemicals produce mild or no lesions (LPG VII, VIII). For intermediate grades of NR activity (NRG B-F), the relationships are more complex: PPAR, PXR and SR activators (NRG B) produced stage (iii) lesions (neoplastic) in both species (LPG I, V); most CAR and PXR (NRG D) activators produced stage (ii) lesions but some were also hepatocarcinogens; AhR, CAR and PXR activators (NRG E) were mostly mouse hepatocarcinogens.

More importantly, the association of LPG I through VIII with NRG A through G, shown in Figure 4(c) is statistically significant with a p-value of 0.013 using Fisher's exact test. There is greater than 95% confidence that the observations on human nuclear receptor activity and rodent cancer lesion progression are not by chance alone.

## Discussion

Chemical-induced activation of NR has been evaluated previously using HTS [3], [39], [40] but ToxCast is the largest publicly available data set in terms of chemicals (309), number and diversity of NR activities (7), NR assays (54), and associated rodent *in vivo* toxicity data in ToxRefDB [5]. By analyzing the data, we show that these chemicals concurrently activate multiple members of the NR superfamily (NRG) in combinations that have not been possible to systematically describe before. Since the 309 chemicals may not be a representative sample of all environmental pollutants and because we did not measure all NR, it is difficult to say whether these nuclear receptor groups (NRG) are universal. Yet our findings were generally consistent with what is known about the NR activities for some chemicals.

Histopathologic observations in the liver have been also been organized by severity for acute [4] and chronic injury in the past. In our analysis, we integrated diverse phenotypic observations of disease symptoms progressing from adaptive changes to neoplastic lesions. In addition, we also summarized cancer progression data across rat and mouse to contrast subtle differences in the severity of adverse chronic outcomes. While this simplified the computational analysis of phenotypic data, it also represents three possible limitations. First, all stages of lesion progression may not have been observed at the terminus of a chronic bioassay. Second, we did not consider the impact of gender and developmental stages, which can be quite important in chemical carcinogenesis. Third, we did not use information about the concentration at which lesions were observed. This may be especially problematic for chemicals that are dose limited (e.g. acetylcholinesterase inhibitors, many of which are in the current data set), so that doses that might lead to liver toxicity are never reached.

Finding robust relationships in real datasets is difficult because measurements can be noisy or irrelevant, and observations can be uninformative. While our analysis is not immune from these issues we tried to mitigate their influence in two main ways. First, we combined data on disparate molecular assays into an aggregate measure of NR activity. The accuracy of this aggregate activity can be demonstrated by the correct categorization of most replicate chemicals into the same NRG (see Table 1.), despite differences in NR assay profiles. Second, we grouped sparse observations on histopathologic effects into three stages of lesion severity in hepatocarcinogenesis. By independently organizing the observations at these disparate biological scales, we found coherent bioactivity profiles in relation to pathologic states.

Our findings have three main implications for toxicity testing. First, it may be important to screen chemicals for multiple NR activities for assessing the hazard of non-genotoxic liver cancer. Second, the visualization in Figure 4 suggests a possible approach for interpreting disparate NR assays in the context of rodent liver cancer severity, and also shows the uncertainties in using these data for chemical prioritization. Third, NR activation by environmental chemicals may be more conserved between rodents and humans than previously believed [42]. This is corroborated partly by comparison with the literature and also by similarities between the aggregate activities of nuclear receptors across chemicals, which appear to recapitulate their evolutionary relationships (Figure 3(b)). Such a gradual functional divergence in the NR superfamily is consistent with protein evolution [43] but it may also lead to conservation of NR activities between rodents and humans. Relating these responses to divergent phenotypic outcomes, however, requires a deeper understanding of non-genotoxic pathways to cancer.

Chronic animal testing is infeasible for the many thousands of chemicals in commerce, but it is currently the gold-standard for estimating human cancer risk. The EPA ToxCast program is systematically assessing the value of high-throughput technologies for screening environmental chemicals' ability to impact toxicity pathways leading to human diseases such as cancer. Our objective was to develop a tool for efficiently stratifying thousands of environmental chemicals based on their perturbation of events leading to adverse outcomes. Here we focused on liver cancer because it is frequently observed across the 309 ToxCast chemicals, and on NR activity since it is a putative key event in rodent carcinogenesis. Through a unique analysis of these data we found that human NR activity profiles for the chemicals stratified their liver cancer lesion progression in rodents. This relationship between the *in vitro* molecular assays to *in vivo* rodent outcomes identifies putative mode of action, advances our understanding of nuclear receptor interactions with environmental chemicals, and suggests approaches for efficient tiered testing for environmental carcinogens.

## Methods

### Multiplexed Gene Expression in Human Primary Hepatocytes

This is a collection of multiplexed gene expression assays focused on Phase I and II xenobiotic metabolizing enzymes and transporters. Human primary cell cultures were treated with chemicals at 5 concentrations (0.004-40 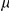
M) for 6, 24 and 48 hr. Concentration- and time-response profiles of chemicals were measured by the expression of key nuclear receptor target genes, activities of CYP1A enzymes (EROD), and cell morphology. Fourteen gene targets were monitored by quantitative nuclease protection assay including: six representative cytochrome P-450 genes, four hepatic transporters, three Phase II conjugating enzymes, and one endogenous metabolism gene involved in cholesterol synthesis. The target genes associated with nuclear receptor pathways are as follows: 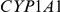 and 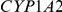 with 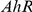; 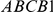, 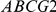, 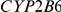, 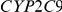, 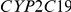 and 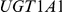 with 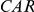; 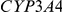, 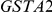, 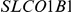 and 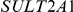 with 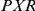; 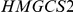 with 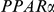; and 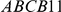 with 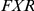. Assays were run in primary human hepatocyte cultures by CellzDirect Invitrogen Inc. (Durham, NC), in collaboration with EPA.

### Multiplexed Transcription Reporter Assays

A multiple reporter transcription unit (MRTU) library consisting of 48 transcription factor binding sites was transfected into the HepG2 human liver hepatoma cell line [20]. In addition to the cis-acting reporter genes (CIS), a modification of the approach was used to generate a trans-system (TRANS) with a mammalian one-hybrid assay consisting of an additional 25 MRTU library reporting the activity of nuclear receptor super-family members. Based on an initial cytotoxicity screen, the maximum tolerated concentration (MTC) was derived as one-third the calculated IC50 or, if no IC50 was determined, the MTC was set to 100 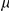
M. Chemicals were then tested in the CIS and TRANS assays at seven concentrations starting at the MTC and followed by three-fold serial dilutions. These assays were performed by Attagene Inc. (Morrisville NC) under contract to EPA.

### Cell-free HTS Assays

These are a collection of biochemical assays measuring binding constants and enzyme inhibition values. Chemicals were initially screened at a single concentration in duplicate wells at a concentration of 10 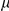
M for cytochrome P-450 assays and 25 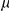
M for all others. Chemicals that showed significant activity were then run in concentration response format, from which an AC50 value was extracted. For concentration response, 8 concentrations were tested in the ranges 0.00914-20 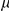
M for cytochrome P-450 assays and 0.0229-50 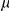
M for other assays. These assays were run by Caliper Life Sciences (Hanover, MD) under contract to EPA. Short assay descriptions are available at: http://www.caliperls.com/products/contract-research/in-vitro/.

### Cell-based HTS Assays

These assays measure binding constants and enzyme inhibition values for nuclear receptors. The targets include 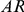, 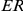, 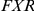, 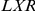, 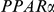, 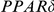, 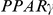, 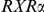, 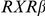 and 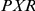. Each of the nuclear receptor targets was measured in agonist mode. Assays were run at the NIH Chemical Genomics Center (Rockville, MD).

### In vitro data

All data used in this analysis are publicly available from the ToxCast website (www.epa.gov/toxcast). The analysis was conducted using the R statistical language (www.r-project.org). For each chemical 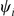, and assay 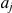 combination we derived either the AC50 (50% maximal activity concentration) or LEC (Lowest Effective Concentration) in 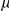
M denoted as, 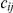. All chemicals are provided in Dataset S3 and all assays are given in Dataset S1. The procedure for evaluating the quality of 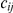 are described in the supplementary methods (Text S1) and all assay results are provided in Dataset S2. In order to facilitate comparison across the assays the 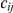 were transformed by the formula, 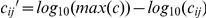 where 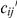 represents a the potency on an ascending scale, and 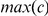 is the maximum concentration (lowest potency) across the data set.

### Aggregating assay results

The aggregate NR activity 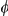, where 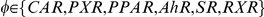, was calculated using assays 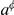. The aggregate scaled NR activity score for each chemical 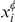 was calculated as the average concentration value across the assays, scaled by the maximum value across all chemicals using Equation 1.
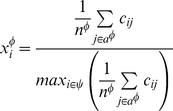
(1)


The number of assays in 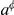 is given by 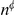. Hence, the complete NR activity of each chemical was defined as a vector using Equation 2.
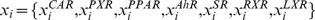
(2)


The NR activities of all chemicals were expressed as a matrix 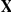, where the rows are chemicals (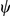) and columns are the NR activities (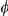).

### Lesion Progression in Hepatocarcinogenesis

*In vivo* rat and mouse long-term histopathology outcomes were extracted for 171 chemicals from ToxRefDB and organized by severity into three stages [5] including: (i) non-proliferative, (ii) putative pre-neoplastic and (iii) neoplastic lesions. For each chemical, the incidence of hepatic tissue lesions was summarized across rat and mouse species as a 6-dimensional cancer lesion progression vector (LPV), which is depicted in Figure 2(a). The resulting 37 unique LPV are shown in Figure 2(b). These LPV were clustered by similarity (described below) into eight lesion progression groups (LPG). The LPG are visualized as a heatmap in Figure 2(c) and denoted by uppercase Roman numerals. The colors of each cell in this heatmap are proportional to the number of chemicals in the LPG that induce a specific lesion type.

### Statistical tests

In addition to the two-sided t-test, 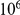 permutations were carried out to empirically estimate the significance (type-I error) of reported univariate statistics. The p-values calculated in this way were adjusted for multiple comparisons using the false discovery rate [44] (FDR) correction. The statistical significance of associations between NRG and LPG were evaluated using Fisher's exact test.

### Clustering

Hierarchical clustering was carried out using the Euclidean distance metric and Ward's minimum variance method for agglomeration. The LPV and the NR data sets were partitioned using k-means [45] clustering for 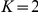 to 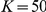, the resulting partitions 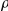 were analyzed using the silhouette method [46], and the value of k was selected by examining the average cluster width. This procedure was used to partition chemicals into groups of NR activity, 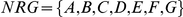, and groups of cancer lesion progression, 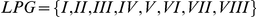.

### Cluster stability

The assignment of chemicals to NRG and LPG was evaluated by a cluster stability score, which was calculated using a subspace sampling approach [47]. A subspace, 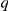, of the data was defined by randomly selecting 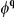 assays and 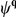 chemicals, where 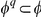 and 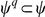. For the chemicals 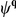, the aggregate scaled activities across 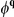 were calculated using Equation 1 to create the subspace data matrix 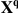. The matrix 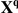 was analyzed by k-means clustering (see above), and chemicals 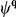 were then assigned to subspace clusters 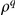. The partitions in 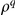 were matched with the partitions 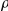 (from the complete data set) based on the maximum number of common members. That is, 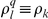 when 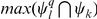 and 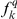 is the fraction of chemicals from 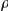 in 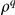. The subspace sampling was conducted 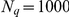 times and the quality 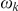 of the partition 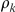 was calculated as 
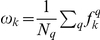
.

### Disclaimer

The United States Environmental Protection Agency through its Office of Research and Development reviewed and approved this publication. Reference to specific commercial products or services does not constitute endorsement.

## Supporting Information

Text S1 Supplementary Methods.
(0.03 MB DOC)

Dataset S1 Description of assays.
(0.01 MB TXT)

Dataset S2 Assay results for each chemical.
(0.06 MB TXT)

Dataset S3 Description of all chemicals used in the analysis.
(0.05 MB TXT)

Dataset S4 Nuclear Receptor Groups for chemicals negative for any liver toxicity.
(0.00 MB TXT)
