# Supplementary material for: Using Nuclear Receptor Activity to Stratify Hepatocarcinogens
Source: PLoS One. 2011 Feb 14;6(2):e14584. doi: 10.1371/journal.pone.0014584 (PMC3038857; doi:10.1371/journal.pone.0014584)
Supplement: Text S1 — Supplementary Methods. (0.03 MB DOC) [file pone.0014584.s001.doc]

**Text S1: Supplementary Methods**

**Shah et al., “Using Human Nuclear Receptor Activity to Stratify Rodent Hepatocarcinogens”**

**Supplementary Methods**

**Data Availability**: All of the data described in this article is available from the EPA ToxCast web site: <http://www.epa.gov/toxcast>. The complete set of AC50/LEC values are contained in a series of 9 files packaged into a zip files with an accompanying README describing the contents.

**Chemical Library**: This study employed a chemical library of 320 substances that are listed online(U.S. EPA 2008). The chemical structures are given in a structure definition file (SDF). There are 309 unique chemical structures, along with three sets of triplicate samples (same source/lot/batch) and 5 sets of duplicate samples (obtained from different commercial sources) used for assessing assay replicability. The majority of these chemicals are current or former food-use pesticide active ingredients designed to be bioactive, while the rest are industrial chemicals of environmental relevance. Chemical information was quality reviewed and structure-annotated within the DSSTox database project (for more information on DSSTox quality review procedures and standard chemical fields, see: <http://www.epa.gov/ncct/dsstox>). Chemicals comprising the ToxCast Phase-I library were commercially procured and plated by BioFocus DPI (South San Francisco, CA). Supplier-provided certificates of analysis indicated purity >97% for the large majority of chemicals (87%), and >90% purity for all but a few instances of technical grade or known mixtures. Follow-up analysis of an original solution plate by BioFocus DPI using LC/MS (liquid chromatography mass spectrometry), subsequent to assay screening, has confirmed mass identification, stability, and purity in excess of 90% for over 77% of the chemical library, with follow-up confirmation studies using GC/MS (gas chromatography MS) planned for chemicals in the remaining set. A QC score associated mapped to chemical sample and solution IDs will be provided on the ToxCast website in association with publication of the assay results.

**AC50/LEC Calculation**: For assay sets where saturating behavior was expected, concentration-response curves were fit to a 3 or 4 parameter Hill function and an AC50 (half maximal activity concentration)(Inglese et al. 2006) value was derived. For assay sets where saturating behavior was not typically seen (such as in RNA expression assays) a Lowest Effective Concentration (LEC) was identified as the lowest concentration at which there was a statistically significant change from the concurrent negative control. LEC values were typically at lower concentrations than AC50 values because, even in the case of saturating behavior, the LEC will occur before the AC50 and can be determined even if 50% activity is never reached. Assay sets for which the characteristic concentration is an LEC tended to have more active chemicals per assay and to have lower characteristic concentrations on average. For cell-based assays, cytotoxicity can cause artifactual appearance of specific cellular phenotypes. Therefore, specific chemical-assay combinations were called inactive if the AC50/LEC was equal to or greater than the lowest cytotoxic concentration measured in the same cell line.

**Cell-free HTS Assays**: A collection of biochemical assays measuring binding constants and enzyme inhibition values. There are a total of 239 endpoints. Chemicals were initially screened at a single concentration in duplicate wells at a concentration of 10 μM for CYP450 assays and 25 μM for all others. Chemicals-assay pairs that showed significant activity were then run in concentration response format, from which an AC50 value was extracted. For concentration response, 8 concentrations were tested in the ranges 0.00914-20 μM for CYP assays and 0.0229-50 μM for non-CYP assays. These assays were run by Caliper Life Sciences (Hanover, MD) under contract to EPA. Short assay descriptions are available at <http://www.caliperls.com/products/contract-research/in-vitro/>. Values for all endpoints were imported into GeneData Screener (Basel, Switzerland) and normalised to solvent controls from the same plate as the tested compounds and expressed as percent of neutral control. Concentration-response curves were fit in GeneData Screener (Basel, Switzerland) Condeseo module using the Hill equation and AC50 values were determined. Data were fit to 4 parameter Hill model as first choice; secondarily a 3 parameter Hill fit was used, generally with a fixed top of curve at 100% of positive control. Detailed description of the methods, data and analysis methods for this dataset are described in a separate publication.

**Cell-based HTS Assays**: These assays measuring binding constants and enzyme inhibition values for nuclear receptors. The targets include AR, ER, FXR, LXR, PPARα, PPARδ, PPARγ, RXRα, RXRβ and PXR. Each of the nuclear receptor targets was measured in both agonist and antagonist mode, but the antagonist data is still undergoing analysis due to possible interference between cytotoxicity and assay activity, so antagonist mode data were not used in the present analysis. Assays were run at the NIH Chemical Genomics Center (Rockville, MD) as part of the Tox21 collaboration. Normalised data from the NCGC were imported into GeneData Screener (Basel, Switzerland) Condeseo Module and fit to 4 parameter Hill model as first choice; secondarily a 3 parameter Hill fit was used, generally with a fixed top of curve at 100% of control unless good indication of partial agonism. Minimum 25% of control efficacy required to consider the chemical to be active. The R^2^ was required to be ≥0.5. Values where only the highest concentration exceeded 50% activity were excluded to eliminate weak or false positives. Detailed description of the methods, data and analysis methods for this dataset will be presented in a separate publication.

**Multiplexed Gene Expression in Human Primary Hepatocytes**: This is a collection of multiplexed gene expression assays focused on Phase I and II metabolic enzymes and transporters. Concentration- and time-response profiles of chemicals are measured by changes in 1) the expression of key nuclear receptor target genes, 2) CYP1A enzymatic activity (EROD), and 3) cell morphology. Fourteen gene targets were monitored by quantitative nuclease protection assay: six representative cytochrome P450 genes, four hepatic transporters, three Phase II conjugating enzymes, and one endogenous metabolism gene involved in cholesterol synthesis. Cells were exposed at 5 concentrations (0.004-40 μM) for 6, 24 or 48 hr. Gene targets are sentinels for five nuclear receptor signaling pathways: AhR, CAR, PXR, FXR, and PPARα. All gene are associated with nuclear receptor pathways: CYP1A1 and CYP1A2 with AHR; ABCB1, ABCG2, CYP2B6, CYP2C9, CYP2C19 and UGT1A1 with CAR; CYP3A4, GSTA2, SLCO1B1 and SULT2A1 with PXR; HMGCS2 with PPARA; and ABCB11 with FXR. Assays were run in primary human hepatocyte cultures (LeCluyse et al. 2005) by CellzDirect Invitrogen Inc. (Durham, NC), in collaboration with EPA. Detailed description of the methods, data and analysis methods for this dataset will be presented in a separate publication.

**Multiplex Transcription Reporter Assays**: A multiple reporter transcription unit (MRTU) library consisting of 48 transcription factor binding sites was transfected into the HepG2 human liver hepatoma cell line as previously described (Romanov et al. 2008). In addition to the cis-acting reporter genes (CIS), a modification of the approach was used to generate a trans-system (TRANS) with a mammalian one-hybrid assay consisting of an additional 25 MRTU library reporting the activity of nuclear receptor super-family members. Based on an initial cytotoxicity screen, the maximum tolerated concentration (MTC) was derived as one-third the calculated IC50 or, if no IC50 was determined, the MTC was set to 100 μM. Chemicals were then tested in the CIS and TRANS assays at seven concentrations starting at the MTC and followed by three-fold serial dilutions. These assays were performed by Attagene Inc. (Morrisville NC) under contract to EPA. Detailed description of the methods, data and analysis methods for this dataset will be presented in a separate publication.
